# Efficacy and Safety of Letibotulinum Toxin A for the Treatment of Dynamic Equinus Foot Deformity in Children with Cerebral Palsy: A Randomized Controlled Trial

**DOI:** 10.3390/toxins9080252

**Published:** 2017-08-18

**Authors:** Hyun Jung Chang, Bo Young Hong, Sang-Jee Lee, Soyoung Lee, Joo Hyun Park, Jeong-Yi Kwon

**Affiliations:** 1 Department of Physical Medicine and Rehabilitation, Samsung Changwon Hospital, Sungkyunkwan University School of Medicine, Changwon 51353, South Korea; reh.chj@gmail.com; 2 Department of Rehabilitation Medicine, St. Vincent’s Hospital, College of Medicine, The Catholic University of Korea, Suwon 16247, South Korea; byhong@catholic.ac.kr; 3 Department of Rehabilitation Medicine, Daejeon St. Mary’s Hospital, College of Medicine, The Catholic University of Korea, Daejeon 34943, South Korea; upperlimb@catholic.ac.kr; 4 Department of Rehabilitation Medicine, Keimyung University, Dongsan Medical Center, Daegu 41931, South Korea; sylee@dsmc.or.kr; 5 Department of Rehabilitation Medicine, Seoul St. Mary’s Hospital, College of Medicine, The Catholic University of Korea, Seoul 06591, South Korea; drpjh@catholic.ac.kr; 6 Department of Physical and Rehabilitation Medicine, Samsung Medical Center, Sungkyunkwan University School of Medicine, Seoul 06351, South Korea

**Keywords:** cerebral palsy, spasticity, botulinum toxin, letibotulinum toxin, onabotulinum toxin

## Abstract

The objective of this clinical trial was to compare the efficacy and safety of letibotulinum toxin A and onabotulinum toxin A for improving dynamic equinus foot deformity in children with cerebral palsy (CP). In total, 144 children with spastic CP who had dynamic equinus foot deformity were assigned randomly to the Botulax group (injection of letibotulinum toxin A) or the Botox group (injection of onabotulinum toxin A). The Physician’s Rating Scale (PRS), ankle plantar flexor spasticity using the Modified Tardieu Scale, the Gross Motor Function Measure (GMFM)-88, and the GMFM-66 were completed before injection and at 6, 12, and 24 weeks after injection. The PRS responder rate was 60.27% in the Botulax group and 61.43% in the Botox group at 12 weeks after treatment, and the lower limit of the 95% confidence interval for the between-group difference in responder rates was −17.16%, higher than the non-inferiority margin of −24.00%. The clinical efficacy and the safety profiles of the groups did not significantly differ. The results suggest that injection of letibotulinum toxin A is as effective and safe as that of onabotulinum toxin A for the treatment of dynamic equinus foot deformity in children with spastic CP.

## 1. Introduction

Cerebral palsy (CP), which is characterized by movement and posture disorders caused by non-progressive lesions in the developing brain, is the most common developmental disorder associated with lifelong motor impairment and disability [1,2]. Spasticity is a major impairment in children with CP, affecting approximately two-thirds of this population [3]. Equinus is a common gait abnormality in children with CP, resulting from spasticity of the ankle plantar flexor [4]. 

Since the first reports in the early 1990s, botulinum toxin type-A (BoNT-A) has been used for the management of spasticity in the lower limbs of children with CP [5,6]. BoNT-A injections decrease spasticity locally by interfering with cholinergic transmission at the neuromuscular junction [7]. BoNT-A has been established as an effective treatment for the management of spastic equinus, as it improves gait, goal attainment, and function [8]. 

Various BoNT-A products are used for the management of spasticity, including onabotulinum toxin A (Botox, Allergan Inc., Irvine, CA, USA), abobotulinum toxin A (Dysport, Ipsen Ltd., Slough, Berkshire, UK), letibotulinum toxin A (Botulax, Hugel Inc., Chuncheon, Korea), and incobotulinum toxin A (Xeomine, Merz Pharmaceutical, Frankfurt, Germany) [9,10,11]. Each BoNT-A product acts by a similar mechanism but has different components. Thus, it is possible that each product presents different levels of efficacy and safety.

Letibotulinum toxin A is a novel neurotoxin from the *C. botulinum* CBFC26 strain that shows high toxicity in mice [12]. Also, its toxin and 16S rRNA sequences are completely homologous to those of the ATCC 3502 Hall A strain of onabotulinum toxin A. Letibotulinum toxin A has been subjected to additional processes, including enzyme-free purification steps such as protamine sulfate precipitation, to eliminate nucleic acids, and diethylaminoethanol-sepharose chromatography, which could theoretically improve the quality of the product. However, few studies have assessed the efficacy and safety of letibotulinum toxin A in the treatment of spasticity [12]. Also, there has been no report on the effectiveness of letibotulinum toxin A in the management of spasticity in children with CP.

When newly formulated BoNT-A products are manufactured, clinical trials are needed to demonstrate the efficacy and safety for each indication. Generally, studies on the safety and efficacy of BoNT-A have been performed using a comparative design involving onabotulinum toxin A. 

We hypothesized that the efficacy and safety of letibotulinum toxin A were not inferior to those of onabotulinum toxin A. The objective of this clinical trial was to compare the efficacy of letibotulinum toxin A (Botulax) with that of onabotulinum toxin A (Botox) for improving dynamic equinus foot deformity among children with CP, and to record any adverse events in the two groups.

## 2. Results

**Figure 1:**
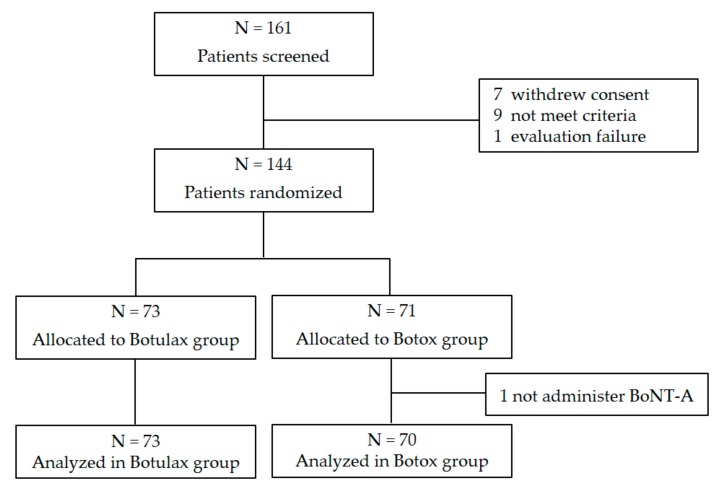
Flow diagram.

In total, 144 subjects participated in the study, with 73 subjects randomly assigned to the Botulax group (injected with letibotulinum toxin A) and 71 assigned to the Botox group (injected with onabotulinum toxin A) (Figure 1).

There were 42 (57.5%) males in the Botulax group and 49 (70.0%) in the Botox group, and there was no statistically significant difference in the gender distribution of the groups. The mean age of subjects was 4.67 years in the Botulax group and 5.44 years in the Botox group; this constituted a statistically significant between-group difference (*p* = 0.0408; Table 1). 

In terms of spastic CP types, unilateral CP was reported in 24 (32.9%) subjects, and bilateral CP in 49 (67.1%) in the Botulax group; in the Botox group, unilateral CP was reported in 24 (34.3%), and bilateral CP was reported in 46 (65.7%). There was no statistically significant difference in the distribution of CP types between the two groups.

For BoNT injection history, 48 subjects (65.8%) were injected with BoNT-A, and 25 subjects (34.25%) were de novo patients in the Botulax group. In the Botox group, 47 subjects (67.1%) had a history of BoNT-A injection, and 23 subjects (32.9%) had never been injected with BoNT before screening. In both groups, there was no subject with BoNT-B injection history, and there was no statistically significant between-group difference in the distribution of BoNT injection history. For subjects with BoNT-A injection history, there was no subject with a previous history of hypersensitivity to BoNT-A. The mean number of BoNT-A injection was 2.6 times in the Botulax group and 3.06 times in the Botox group, which showed no statistically significant difference between groups.

The mean doses of botulinum toxin injected in each group were 100.0 ± 32.9 U in the Botulax group and 106.1 ± 39.4 U in the Botox group. There was no significant difference between the two groups (*p* = 0.497).

### 2.1. Physician’s Rating Scale (PRS)

#### 2.1.1. Responder Rate

The responder rate (proportion of subjects determined to be responders) was 60.27% (44/73 subjects) in the Botulax group and 61.43% (43/70 subjects) in the Botox group at 12 weeks after BoNT-A injection, and the lower limit of the 95% confidence interval (CI) for the difference in the responder rate (−1.15%) between the Botulax group and the Botox group was −17.16%, which was higher than the non-inferiority margin of −24.00% (Table 2) [13]. 

#### 2.1.2. Mean Score

**Figure 2:**
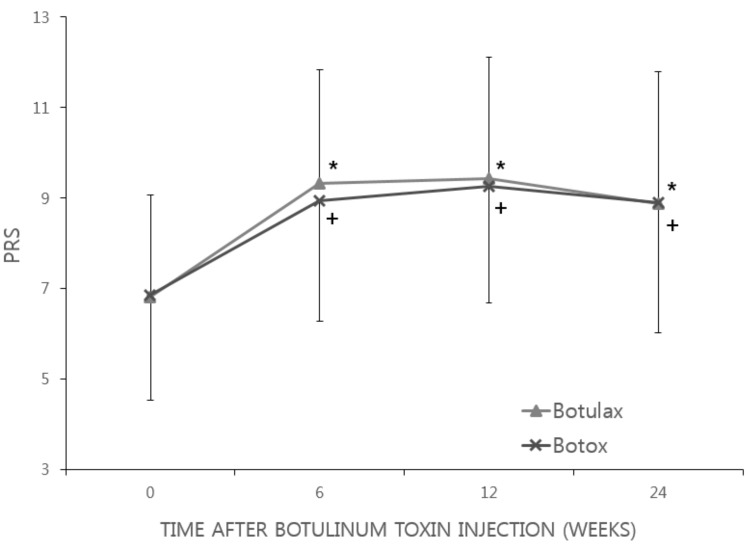
Changes in Physician’s Rating Scale (PRS). In both the Botulax group and Botox group, PRS score was significantly increased and maintained until 24 weeks after BoNT-A injection. The error bar indicates the standard deviation. * Statistically significant when compared between baseline data and post-injection data in the Botulax group; + Statistically significant when compared between baseline data and post-injection data in the Botox group.

The mean PRS score in the Botulax group was 6.82 at baseline, 9.33 at week 6, 9.43 at week 12, and 8.88 at week 24; these figures were 6.85, 8.95, 9.25, and 8.89, respectively, in the Botox group, suggesting that the PRS score increased from the baseline to each time point in both groups, and the changes were statistically significant (*p* < 0.0001; Figure 2). There was no statistically significant difference between the two groups in terms of changes in the PRS scores at 6, 12, or 24 weeks after BoNT-A injection.

### 2.2. Modified Tardieu Scale (MTS)

**Figure 3:**
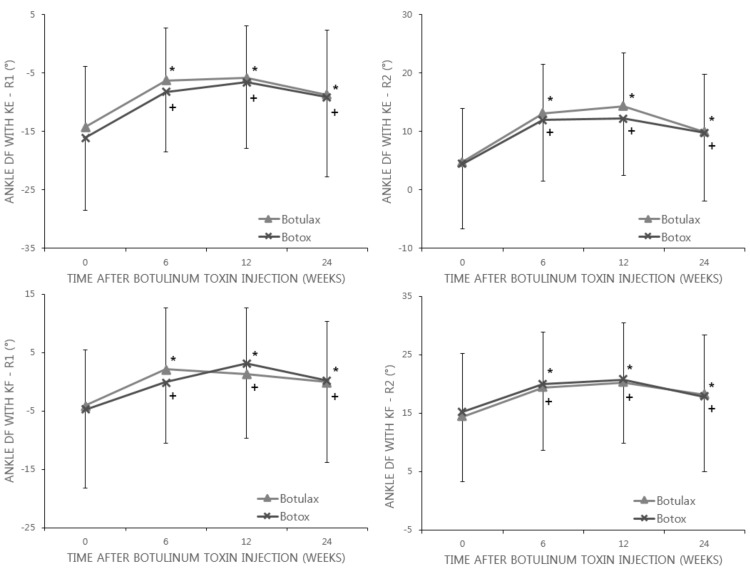
Changes in the Modified Tardieu Scale (MTS) of ankle dorsiflexion (DF) with knee flexion (KF) and knee extension (KE) states. In both the Botulax group and Botox group, ankle DF angles were significantly increased and maintained until 24 weeks after BoNT-A injection. The error bar indicates the standard deviation. * Statistically significant when compared between baseline data and post-injection data in the Botulax group; + Statistically significant when compared between baseline data and post-injection data in the Botox group.

The mean R1 of ankle dorsiflexion with knee extension were −14.19° at baseline, −6.24° at week 6, −5.83° at week 12, and −8.74° at week 24 in the Botulax group; these figures were −16.06°, −8.24°, −6.59°, and −9.14°, respectively, in the Botox group. And the mean R2 of ankle dorsiflexion with knee extension were 4.76° at baseline, 13.16° at week 6, 14.33° at week 12, and 9.87° at week 24 in the Botulax group; these figures were 4.48°, 11.96°, 12.19°, and 9.75°, respectively, in the Botox group, which demonstrated that the mean R1 and R2 of ankle dorsiflexion with knee extension changed in a positive direction from the baseline to 6, 12, and 24 weeks after BoNT-A injection in both groups. Although the within-group difference for each change was statistically significant (*p* < 0.0001), the between-group difference was not statistically significant (Figure 3).

The mean R1 of ankle dorsiflexion with knee flexion were −4.12° at baseline, 2.09° at week 6, 1.34° at week 12, and −0.08° at week 24 in the Botulax group; in Botox group, these values were −4.76°, −0.08°, 3.08°, and 0.24°, respectively. Also, the mean R2 of ankle dorsiflexion with knee flexion were 14.31° at baseline, 19.40° at week 6, 20.21° at week 12, and 18.15° at week 24 in the Botulax group; in Botox group, these values were 15.19°, 20.01°, 20.71°, and 17.73°, respectively. These alterations showed that the mean R1 and R2 of ankle dorsiflexion with knee flexion changed in a positive direction from baseline to each time point in both groups. Although the within-group difference for each change was statistically significant (*p* < 0.001), the between-group difference was not statistically significant (Figure 3).

### 2.3. Gross Motor Function Measure (GMFM)

The means of the GMFM-88 and GMFM-66 increased from baseline to each time point in both groups. Although the within-group difference for each change was statistically significant (all *p* < 0.0001), the between-group difference was not statistically significant (Table 3). 

### 2.4. Safety Profiles

In total, 152 adverse events were reported in 54 subjects in the Botulax group (73.97%), and 130 events were reported in 45 subjects in the Botox group (64.29%) after injection, and there was no significant between-group difference in the incidence of adverse events (*p* = 0.2096; Table 4). As frequent adverse events in the Botulax group, nasopharyngitis was the most common with 68 events in 32 subjects (43.84%), followed by bronchitis with 19 events in 11 subjects (15.07%). In the Botox group, nasopharyngitis was the most common with 61 events in 27 subjects (38.57%), followed by bronchitis with 16 events in nine subjects (12.86%).

The causal relationship with the investigational product was assessed as ‘definitely related’, ‘probably related’, ‘possibly related’, ‘probably not related’, ‘definitely not related’, or ‘unknown’; of these, adverse events other than those evaluated as ‘probably not related’ or ‘definitely not related’ were classified as adverse drug reactions for which the causal relationship with the investigational product could not be ruled out. There was no adverse drug reaction in the Botulax group, whereas two adverse drug reactions (two subjects, 2.86%) of urticaria and dysphonia were reported in the Botox group. There was no statistically significant difference between the two groups in the incidence of the acute adverse events.

As acute adverse events that occurred within 30 min after BoNT-A injection, two acute adverse events of pain and erythema at the injection site occurred in one subject (1.37%) in the Botulax group, which were mild in severity and completely recovered without sequelae. No acute adverse event was reported in the Botox group. There was no statistically significant difference between the two groups in the incidence of the acute adverse events (Table 4).

In terms of serious adverse events that rendered performance of the activities of daily living impossible or required hospitalization, 10 subjects reported 14 events. In the Botulax group, five events occurred in four subjects (5.48%), including intussusception, tonsillar hypertrophy, asthma, bronchitis, and pharyngotonsillitis, and those events completely recovered without sequelae. In the Botox group, nine events in six subjects (8.57%) were reported, including acute tonsillitis, osteochondrosis, meningitis, and six events of pneumonia; they were all moderate and completely recovered without sequelae. There was no statistically significant difference between the two groups in the serious adverse events incidence (Table 4).

## 3. Discussion

This randomized, double-blind, controlled trial showed that BoNT-A injection at the gastrocnemius muscle improved dynamic equinus foot deformity in children with spastic CP and that the effectiveness and safety of letibotulinum toxin A were not inferior to those of onabotulinum toxin A. 

These results are similar to those of previous studies on the effect of BoNT-A injection on dynamic equinus in CP. In our study, the overall responder rate after letibotulinum toxin A or onabotulinum toxin A injection was 60.84% (87/143). Kim et al. reported that the overall responder rate after BoNT-A injection was 54.6% [9]. Also, Koman et al. reported that repeated BoNT-A injection improved gait patterns in children with CP, and the responder rate was 55% at the 1-year follow-up [14]. The PRS score and range of motion in ankle dorsiflexion were improved at 6 and 12 weeks after injection, and they decreased slightly at 24 weeks. These results were similar to those of a previous study that suggested the effects of BoNT-A injection on gait lasted, on average, for 3–5 months [14]. However, the GMFM continued to increase until 24 weeks after BoNT-A injection. Boyd and Graham reported that CP children gained significant functional improvement that lasted up to 1.5 years after BoNT-A injection [15]. We suggest that the improvement in gait pattern and range of motion in ankle dorsiflexion after BoNT-A injection for dynamic equinus foot deformity was maximal at 3 months and lasted up to 6 months, and the functional increase continued until 6 months after injection. These effects did not significantly differ between letibotulinum toxin A and onabotulinum toxin A.

The subjects in the Botulax group were significantly younger than those in the Botox group. However, the age stratification (2–4 years; 5–7 years; 8–10 years) showed that there was no significant difference in age category between two groups (*p* = 0.202). Also, we conducted secondary analyses to assess whether age affected the effectiveness of the BoNT-A injection (to be published separately). Multiple regression analyses showed that age was not a significant factor that affected the efficacy of BoNT-A injection. Jang et al. also reported that age was not associated with the effects of BoNT-A injection on muscle tone, passive range of motion, or gait pattern [16].

Our study showed an incidence of global adverse events in the whole cohort of 69.2% (99/143). This incidence seems to be higher than that reported in other studies [9,10,11]. However, the age range of the subjects in this study was 2–10 years (mean age, 5.0 years). The age range of the subjects included in the study of Carraro et al. was 3–18 years, and that of those in the study of Delgado et al. was 2–17 years. Given that the incidence of illness might be higher in younger children, it is important to note that the subjects in this study were younger than the subjects included in previous studies. Also, the follow-up period of this study was 24 weeks, which is longer than that of previous studies (12 weeks). Thus, there was more opportunity for a disease to occur regardless of BoNT-A injection due to the longer period. Indeed, seven among 14 serious adverse events in whole cohort occurred between 3 and 6 months after BoNT-A injection. Another reason for the result could be because the criteria for reporting adverse events differed. Indeed, more than half of the adverse events were grade 1, meaning that they did not interfere with normal daily life to such an extent that the subject hardly noticed them. Another reason could be that the barriers to access to healthcare in Korea are very low. 

Only 0.7% of global adverse events were injection-related, according to an investigation of the causal relationship between adverse events and drugs. This is a much smaller incidence than those reported in previous studies (>2% of subjects) [11]. Furthermore, no adverse event in the Botulax group was definitely or probably related to BoNT-A injection. Also, no serious adverse event in either group was definitely or probably related to BoNT-A injection. This was because the BoNT-A dosage used in this study was less than those in previous studies. Indeed, another study in which they injected doses of BoNT-A similar to those used in this study reported no adverse events related to the treatment [9]. 

This study has several limitations. There was no placebo group, which is needed to confirm the net effect of a study drug. However, treatment with BoNT-A injections for children with CP has already been established. Thus, injection with a placebo was not considered to be ethically appropriate. Also, it was not permitted to use variable doses of BoNT-A according to spasticity, which could lead to optimal results. The dose used in our protocol might not have been sufficient in some children. Further study is needed to identify the most appropriate dose for the severity of spasticity. 

## 4. Conclusions

Injection of letibotulinum toxin A was shown to be as effective and safe as that of onabotulinum toxin A for the treatment of dynamic equinus foot deformity in children with spastic CP. The results of this study are expected to provide physicians with another choice for the treatment of spasticity in children with CP.

## 5. Materials and Methods

This study was designed as a double-blind, randomized, active-controlled comparative, multicenter-designed, phase III clinical trial. The trial was registered at clinicaltrial.gov (NCT01787344), and approved by the Ministry of Food and Drug Safety. The study protocol was also approved by the Institutional Review Boards (IRBs) of the six participating hospitals in the Republic of Korea. The project identification codes, the names of the IRBs, and the date of approval are as follows; DC12BDMT0002 at Daejeon Daejeon St. Mary’s Hospital, College of Medicine, The Catholic University of Korea on 01/30/2012; KC12BDMT0097 at Seoul St. Mary’s Hospital, College of Medicine, The Catholic University of Korea on 02/28/2012; VC12BDMT0033 at St. Vincent’s Hospital, College of Medicine, The Catholic University of Korea on 02/28/2012; 12–43 02.21 at Keimyung University, Dongsan Medical Center on 03/05/2012; SMC2012-02-022-001 at Samsung Medical Center, Sungkyunkwan University School of Medicine on 02/29/2012; 2012-SCMC-009-00 at Samsung Changwon Hospital, Sungkyunkwan University School of Medicine on 03/22/2012. This investigation was carried out following the rules of the Declaration of Helsinki. All subjects gave their informed consent for inclusion before participating in the study.

This study consisted of screening and then injection, followed by a 24-week observation period. Patients who provided written consent to participate in the study were screened and assessed for eligibility as subjects. The inclusion criteria were as follows: (1) children aged between 2 and 10 years; (2) diagnosis of spastic CP (confirmed via magnetic resonance imaging, as necessary); (3) Gross Motor Function Classification System Level I, II, or III; and (4) diagnosis of dynamic equinus foot deformity (with heel lift-up during walking). The exclusion criteria were as follows: (1) received botulinum toxin injection within 3 months before screening; (2) previous history of hypersensitivity to botulinum toxin products or their ingredients; (3) diagnosis of systemic neuromuscular junction disorder; (4) scheduled for surgery on the leg, foot, or ankle or previous history of surgery at the corresponding sites; and (5) leg length discrepancy >5 cm. 

Enrolled subjects were randomly assigned to the Botulax group (injection with letibotulinum toxin A) or the Botox group (injection with onabotulinum toxin A) at a ratio of 1:1. The random assignment code was generated using a block randomization method for each hospital. Those codes were sealed until the trial was completed except in cases where it was inevitably necessary to view the code due to a serious adverse event. Thus, all research staff, clinicians, and participants were unaware of trial group assignments.

After randomization, participants received the corresponding BoNT-A to two sites on the medial and lateral heads of gastrocnemius muscle. In the case of bilateral CP, BoNT-A was administered to both legs at a dose of 6 U/kg body weight (3 U/kg for each leg). In the case of unilateral CP, the BoNT-A was administered to the spastic calf muscle at a dose of 4 U/kg body weight. The investigational products were packaged with same container and label. The personnel responsible for dilution prepared the injection solution with the corresponding BoNT-A apart from injection place. The syringe without apparent difference between two investigational products was handed over to the injector who was in charge of BoNT-A injection. Therefore, blinding of the injectors could be maintained. Also, blinding of the investigators and physical therapist in charge of assessments, patients, and parents were maintained. Local anesthetic cream was applied to the injection site 30 min before injection. The injection was guided with ultrasonography or electrical stimulation according to preference of the injectors in each center. For the detection of the occurrence of acute adverse events, the subject’s status was observed for 30 min after injection of the investigational products.

The subjects visited the study centers at 6, 12, and 24 weeks after administration of the investigational products to undergo efficacy and safety assessments.

### 5.1. Outcome Measures

The primary outcome was measured by the responder rate of the PRS score at 12 weeks after injection. The PRS score represented the dynamic gait pattern during active walking [17]. It consists of six functional components of the human gait cycle, and each component is scored on a scale of 0 to 2 or 3. The six component scores were summed up to yield the PRS score, which ranged from 0 to 14 (0 = worst, 14 = best). It was scored by the investigators at the corresponding visit and recorded using a video camera for more accurate assessment. The responder rate was defined as the percentage of subjects whose PRS scores increased by 2 or more compared with that at baseline. In the case of bilateral CP, the outcome was assessed to be effective if the PRS score for each leg increased by 2 or more. 

The secondary outcomes measured were the responder rate of the PRS score at 6 weeks and 24 weeks after BoNT-A injection, the MTS of ankle dorsiflexion, and the GMFM-88 and GMFM-66.

The MTS is a valid, reliable, and sensitive tool for the assessment of spasticity [18]. Two angles (R1 and R2) were determined in the MTS. The R1 was the angle at which a velocity-dependent catch or clonus was felt during a fast stretch of the muscle. The R2 was the joint angle when the muscle length was at its maximum, using slow passive movement. The neutral position at the ankle joint (angle zero) was the position with the ankle at 90° to the lower leg. Degrees of dorsiflexion from the neutral position were indicated by a positive (+) measurement, whereas degrees of plantarflexion from the neutral position were indicated by a negative (−) measurement. The MTS was applied in both the knee flexion and extension positions.

The GMFM, previously demonstrated to have high levels of validity, reliability, and responsiveness in evaluating motor function and assessing the results of management strategies for children with CP, was used to assess clinical changes in the participants [19]. The GMFM-88 is the original 88-item measure designed to evaluate change in gross motor function over time or with intervention for children with CP. The GMFM-88 is organized into five dimensions: (A) lying and rolling, (B) sitting, (C) crawling and kneeling, (D) standing, and (E) walking, running, and jumping. The levels of each item are explicitly defined and scored on a scale of 0 to 3. Item scores are summed to yield scores for each dimension. Subsequently, GMFM-88 scores were converted into GMFM-66 scores using the Gross Motor Ability Estimator. The GMFM-66 is comprised of subset of the 88 items identified (through Rasch analysis) as contributing to the measure of gross motor function in children with CP. The measurement and the scaling of GMFM-66 is somewhat shorter. We used both the GMFM-88 and GMFM-66. The GMFM measurements were conducted by physical therapists who were trained in and clinically experienced with using the GMFM. 

### 5.2. Statistical Analysis

The purpose of the study was to show that letibotulinum toxin A was not inferior to onabotulinum toxin A using the responder rate. Using the weighted average, the responder rates for onabotulinum toxin A and a placebo were estimated to be 62.5% and 26.5%, respectively [17,20]. We calculated that a minimum sample size of 128 patients would be required to provide 80% power at a 5% significance level, assuming a responder rate of 62.5%. Thus, we determined that the estimated sample size was 144 patients (72 per each group), considering a withdrawal rate of 10%.

All statistical analyses were conducted using the SAS software. Two-sided tests, in principle, were conducted at a statistical significance level of ≤5% to test the significance levels of the differences between the two groups, between the efficacy analysis before and after treatment for each group, and between the safety assessments at different time points. Based on the guidance for industry non-inferiority clinical trials, the margin for the difference in responder rates was set at 24% to ensure that letibotulinum toxin A was as effective as onabotulinum toxin A [13]. Statistical analysis evaluated whether the one-sided 95% CI of the difference in responder rates between letibotulinum toxin A and onabotulinum toxin A was within the −24% non-inferiority margin. Pearson’s χ^2^ test or Fisher’s exact test was used to assess the differences in the responder rate of the PRS at each visit and in the occurrence of adverse events between the groups. For PRS score, the MTS, GMFM-88, GMFM-66, and the mean changes after injection were compared between the two groups using two-sample *t*-tests or Wilcoxon’s signed-rank test, and post-injection data at each visit were compared with baseline data using a paired *t*-test or Wilcoxon’s signed-rank test. 

## Figures and Tables

**Table 1:** Baseline demographic and clinical data (FA set = 143 subjects).

Baseline Characteristics	Botulax Group (N = 73)	Botox Group (N = 70)	*p*-Value
Male/female, n (%)	42 (57.5)/31 (42.5)	49 (70.0)/21 (30.0)	0.1214
Age (years), mean (SD)	4.7 (2.0)	5.4 (2.3)	0.0408 *
2–4 years, n	37	26	0.2020
5–7 years, n	28	31	
8–10 years, n	8	13	
Body weight (kg), mean (SD)	18.98 (6.47)	20.01 (6.98)	0.3406
Type, n (%)			0.9336
Right unilateral	18 (24.7)	17 (24.3)	
Left unilateral	6 (8.2)	7 (10.0)	
Bilateral	49 (67.1)	46 (65.7)	
Previous BoNT-A injection, n (%)	48 (65.8)	47 (67.1)	0.8604
Number of previous BoNT-A injectrion, median (min-max)	2 (1–6)	3 (1–7)	0.146
PRS, median (min-max)	6 (2–13)	6 (3–12)	0.4800
GMFM-88	85.7 (13.9)	85.7 (13.7)	0.7140

FA: Full Analysis, PRS: Physician’s Rating Scale, GMFM: Gross Motor Function Measure. * Statistically significant when compared between Botulax group and Botox group (*p* < 0.05).

**Table 2:** Responder rate in the Physician’s Rating Scale (PRS), n (%).

Assessment Week	Botulax Group (N = 73)	Botox Group (N = 70)	*p*-Value
Post-injection 6 weeks	42 (58.9)	36 (51.4)	0.3688
Post-injection 12 weeks	44 (60.3)	43 (61.4)	0.8876
Post-injection 24 weeks	35 (48.0)	29 (41.4)	0.4334

**Table 3:** Changes in Gross Motor Function Measure (GMFM).

GMFM	Botulax Group (N = 73)			Botox Group (N = 70)			Between-Group Difference *p*-Value
	Mean	±SD	Within-Group Difference *p*-Value	Mean	±SD	Within-Group Difference *p*-Value	
GMFM-88							
Pre-injection (Baseline)	85.71	±13.90		85.67	±13.70		
Post-injection 6 weeks	86.78	±13.22	<0.0001 *	86.85	±13.23	<0.0001 *	
Post-injection 12 weeks	87.53	±12.83	<0.0001 *	87.78	±12.86	<0.0001 *	
Post-injection 24 weeks	88.39	±12.36	<0.0001 *	88.53	±12.46	<0.0001 *	
Change (Week 6)	1.07	±2.37		1.18	±2.94		0.3255
Change (Week 12)	1.82	±3.20		2.11	±3.47		0.0806
Change (Week 24)	2.68	±4.23		2.86	±3.91		0.0803
**GMFM-66**							
Pre-injection (Baseline)	70.50	±13.47		69.59	±11.83		
Post-injection 6 weeks	71.53	±13.58	<0.0001 *	71.13	±12.07	<0.0001 *	
Post-injection 12 weeks	72.45	±13.37	<0.0001 *	72.07	±12.59	<0.0001 *	
Post-injection 24 weeks	73.66	±13.55	<0.0001 *	73.33	±12.99	<0.0001 *	
Change (Week 6)	1.04	±3.01		1.54	±2.56		0.2172
Change (Week 12)	1.95	±3.45		2.48	±3.22		0.2663
Change (Week 24)	3.16	±3.52		3.74	±3.20		0.0921

Change = Post-injection − baseline; * Statistically significant when compared between baseline data and post-injection data.

**Table 4:** Adverse events.

Adverse Event	Botulax Group (N = 73)			Botox Group (N = 70)			*p*-Value
	n (%), [Events]		95% CI	n (%), [Events]		95% CI	
Adverse event	54(73.97),	[152]	[62.38, 83.55]	45(64.29),	[130]	[51.93, 75.39]	0.2096
Adverse drug reaction	0(0.00),	[0]	-	2(2.86),	[2]	[0.35, 9.94]	0.2379
Acute adverse event	1(1.37),	[2]	[0.03, 7.40]	0(0.00),	[0]	-	1.0000
Serious adverse events	4(5.48),	[5]	[1.51, 13.44]	6(8.57),	[9]	[3.21, 17.73]	0.5269
